# A review of the outcomes of rigid medical thoracoscopy in a large UK district general hospital

**DOI:** 10.1515/pp-2020-0131

**Published:** 2020-11-02

**Authors:** Avinash Aujayeb, Karl Jackson

**Affiliations:** Northumbria HealthCare NHS Foundation Trust, Care of Tracy Groom, Cramlington, Northumberland, UK

**Keywords:** mesothelioma, pleural effusion, thoracoscopy

## Abstract

**Objectives:**

Local anesthetic medical thoracoscopy (LAT) is a well-established diagnostic, therapeutic, and preventative intervention in undiagnosed pleural effusions with a high diagnostic sensitivity and low complication rates. There is a large variability in practice. We describe a nine-year experience in a large district general hospital in England.

**Methods:**

Two hundred seventy-five patients had LAT between January 2010 and December 2018. Data on outcomes and complications were obtained from the patients’ notes, electronic records, laboratory, and radiographic findings.

**Results:**

The main diagnoses were malignant pleural mesothelioma (MPM) (n=110, 40%), chronic inflammation/fibrinous pleuritis (77, 28%), lung cancer (26, 9.5%), and breast cancer (16, 6%). LAT failed to diagnose cancer in 7/275 patients (false-negative rate 2.5%, diagnostic sensitivity 97.5%). Out of the 105 patients with chronic inflammation/fibrinous pleuritis or atypical proliferative processes, 21 (20%) were subsequently diagnosed with malignancy. Talcum pleurodesis was performed in 146 patients, and was successful in 86%. Seventy eight (28%) patients had trapped lung; 27 of those had a repeat procedure. The median length of stay was 3.96 days. There was one hospital death (0.3% mortality). Complications of LAT included pleural (3, 1%) and wound infections (4, 1.4%), persistent air leaks (9, 3.2%), subcutaneous emphysema (10, 3.6%), and tumor extension to the access port (1, 0.3%).

**Conclusions:**

In this cohort, LAT was safe, effective, and enabled high diagnostic sensitivity. Further areas of study include optimal sedation and anesthetic pathways and combining LAT with indwelling pleural catheters (IPC).

## Introduction

Local anesthetic medical thoracoscopy (LAT), also known as “pleuroscopy” or medical thoracoscopy (MT) was first performed by Sir Francis Richard Cruise in 1865. Sir Cruise used a cystoscope to visualize the thoracic cavity. He recognized the vast diagnostic and therapeutic possibilities of thoracoscopic and laparoscopic approaches, but also the possibility of trocar-induced injury to internal organs, and the requirement for training [1].

The vast majority of LATs are performed for undiagnosed exudative pleural effusions that are presumed to have a malignant etiology [2]. The cytological yield for malignancy from pleural effusions approaches 60% [3]. However, up to 63% of cytology can remain negative in patients with malignant mesothelioma [4]. The yield from closed pleural biopsies for malignancy is low at 57% [2]. Therefore, there is a necessity for biopsies from a directly visualized tumor on the pleura.

The British Thoracic Society (BTS) produced guidelines in 2010 [2], summarizing experience from eight case-series with a total of 361 patients. These guidelines reported a diagnostic sensitivity of over 90% for malignant pleural disease. Successful pleurodesis occurred in over 80%, and the length of stay post-procedure was 4.6 days.

LAT services in the UK are highly variable [5] concerning preoperative patient preparation, location of the procedure, medication use, and the combination with other procedures (such as insertion of indwelling pleural catheters [IPCs] and adhesiolysis). European guidance recommends that LAT is performed under sedation and/or conscious sedation using fentanyl and midazolam or with titrated propofol dosage [6]. LAT can be performed with rigid or semirigid thoracoscopes.

Northumbria Healthcare NHS Foundation Trust has a well-established pleural service [7] for a large geographical region with a population of 500,000. The service delivers 10 pleural clinics a month and has almost weekly access to theater space for LAT and IPC insertion. Three respiratory consultants who perform and supervise trainees, including a pleural fellow.

## Materials and methods

### Study design

This is a retrospective study of all consecutive patients who underwent MT from 2010 to December 2018 in Northumbria Healthcare NHS Trust. The electronic records were accessed, and the following data as collected: age, sex, histological diagnoses, final diagnoses, complication rates, trapped lung rates, pleurodesis rates, and need for further procedures.

### Perioperative management

Patients are consented by the operator on the day, preassessed by an anesthetist, and then taken to the operating theater. Antibiotics prophylaxis [8] with gentamicin or teicoplanin, and more recently, either flucloxacillin or teicoplanin, is administered at the induction of anesthesia. From 2010 to late 2017, we performed LAT under conscious sedation with midazolam and fentanyl. However, increasing concerns relating to lack of intraoperative analgesia and anesthesia led us to change our practice. Since 2018, an anesthetist provides patient-targeted anesthesia with Propofol and Remifentanil. Oxygen is administered via nasal cannula or face masks to maintain oxygen saturation (SpO_2_) over 90%. Blood pressure, cardiac, and oxygen saturation are continuously monitored. After the procedure, a chest radiograph is performed in recovery, and the patient is transferred back to the ward. Suction can be applied in the presence of incomplete lung re-expansion. Patients are usually discharged when output from the drain is less than 200 mL/day.

### LAT technique

The patients are placed in the lateral decubitus position, and a thoracic ultrasound is performed to mark the spot for incision and insertion of any port. The marked area of the chest is then sterilized and draped. Twenty milliliters (mL) of bupivacaine and adrenaline (epinephrine) injection 0.5% w/v, one in 200,000 is administered into the selected intercostal space. A tract is created with curved forceps, and the pleural membrane pierced with blunt and then sharp ends of the Boutin needle. A single 7-mm trocar and port is then inserted. Air is allowed to enter the pleural cavity to create the requisite artificial pneumothorax. LAT is performed with a rigid thoracoscope (Wolf [Richard Wolf Medical Instrumentation Corp., IL, USA]) or Storz (Karl Storz, Tuttlingen, Germany). Pleural fluid is suctioned. Adhesions are broken down to reach the parietal surface and biopsies taken for histopathology and microbiology examinations as required. About 4  g of graded talc (Steritalc®, Novatech, La Ciotat, France) is then insufflated into the pleural cavity with a catheter and bulb syringe. If there is an obvious nonexpandable lung (adherence of the lung to the parietal membrane), an IPC can be inserted at this time. Routinely, a 24 French gauge Argyll chest tube is inserted at the end of the procedure through the entry port and connected to an underwater drainage bottle.

### Outcome measurement

Outcome was measured in accordance with the BTS 2010 guideline [2]. Empyema, hemorrhage, port site recurrence, bronchopleural fistula, postoperative pneumothorax or air leak, and pneumonia were considered major complications subcutaneous emphysema, minor hemorrhage, operative skin site infection, hypotension during the procedure, raised temperature, and atrial fibrillation were classified minor complications

## Results

A total of 275 patients were identified from the database. The patient characteristics are detailed in Table 1.

**Table 1:** Patients’ characteristics.


M:F ratio	216: 59
Age, years; mean (range)	72 (20–89)
LAT side (right:left)	200:75
Histology	
– Pleural mesothelioma	110 (40%)
– Lung cancer	26 (9.5%)
– Metastatic breast cancer	16 (5.8%)
– Empyema	7 (2.5%)
– Chronic inflammation/fibrinous pleuritis (CFP)	77 (28%)
– Atypical proliferative processes	28 (10%)
– Other diagnoses^a^	10 (3.6%)
– Unknown as biopsy not taken	1 (0.4%)
Total	275

a Metastatic melanoma, thymoma, lymphomas, ovarian cancers, pleural tuberculosis, renal cancer, laryngeal cancer, cancer of unknown primary.

Five patients with atypical changes on histology after LAT had a subsequent cancer diagnosis via either surgical or image-guided biopsies due to ongoing concerns that the clinical and radiological picture warranted further investigations. Two patients with histology showing fibrinous pleuritis had clinical and radiological evidence of cancer (significant weight loss, chest pain, and nodular circumferential pleural thickening on CT scans). These two patients were not investigated further due to a rapidly deteriorating performance status. Thus, LAT failed to diagnose cancer in seven patients out of 275, representing a diagnostic sensitivity of 97%.

The operating notes of those seven false-negative patients were reviewed. There was no video available. All had small effusions on thoracic ultrasound at induction, and the initial pleural tap was feasible in all. Pneumothorax was induced in all, but the views of the pleural surface were impeded by significant adhesions in six. In these patients, considerable time was spent dissecting the adhesions to try to biopsy the parietal lining, possibly preventing accurate biopsies. In the last patient, pleura was described as diffusely abnormal (no photos available), deep biopsies were taken under sufficient vision, but no cancer was diagnosed in the histology.

A group of 105 patients had chronic fibrinous pleuritis or atypical proliferative processes. Since such processes can mask or precede cancer development, we follow-up these patients for two years, as reported elsewhere [9]. The mean time to repeat biopsy was 6.8 months (95% CI 3.14–10.87), and the mean follow-up was 16.7 months (95% CI 14.06–19.46). Out of these 105 patients, 21 (20%) were subsequently diagnosed with malignancy. This was biopsy-proven to be pleural mesothelioma in 10 patients. Eleven patients had a diagnosis of a pleural malignancy based on clinical and radiological progression but did not have repeat biopsies due to low-performance status at the time of reassessment. In 88 patients, malignancy was excluded in the follow-up, and the following diseases diagnosed: asbestos-related benign pleural effusion (BAPE) in 67 patients (63.8%), effusions due to cardiac failure in 12 patients (11.4%), chronic empyema in 10 patients (9.5%), and eosinophilic pleural disease due to concurrent medications in two patients (1.9%). In the remaining 14 patients (13.3%), the histological findings did not allow a specific benign or malignant diagnosis.

Pleurodesis was performed in 146 patients (53.1%). In the absence of trapped lung, pleurodesis was successful in 86%. Success was defined as the patient not requiring another procedure for pleural fluid in the 30 days after a LAT. A group of 78 patients (28%) had a nonexpandable lung. An additional intervention was required in 27/275 patients (9.8%) after LAT. Most of those interventions were the placement of an IPC (n=25). Two patients opted for recurrent therapeutic aspirations. Notably, additional procedures were more frequent (34.6%) in patients with nonexpandable lung.

There was one death after LAT (0.3%). The patient had a chronic renal failure, which was overseen at the preoperative assessment, and he received a one-off prophylactic dose of 5 mg/kg gentamicin. This resulted in an acute on chronic kidney injury requiring admission to intensive care and renal replacement therapy. The patient passed away due to probable pneumonia 36 days after LAT. The investigation of the case showed no causal relationship between any intraprocedural aspects of LAT and the death of the patient. Major complications included pleural infection (n=3, 1%), cellulitis around the operation site (n= 4, 1.4%), air leaks more than five days (n=9, 3.4%), surgical emphysema (n=10, 3.6%), and tumor extension (n=1, 1%). Minor complications were one displaced drain, one skin reaction secondary to the dressing, and one wound leak requiring resuturing in theater. There was no complication related to sedation or anesthesia.

## Discussion

The above retrospective analysis represents data from 275 LAT from a single center over a time of nine years. This study has limitations due to its retrospective nature.

The main finding of our study is a high incidence of malignant pleural mesothelioma (MPM), which fits well with epidemiological data showing a very high rate of pleural mesothelioma in the North-East of England. Over the whole period under investigation, our patient cohort was predominantly elderly and male [7]. The local shipbuilding and construction industry in the early and middle parts of the 20th century widely used asbestos as a building and lagging material [10], and the workforce was predominantly male. The high incidence of MPM is well known by the general practitioners in the area, and approximately 90% of patients were referred from primary care to the pleural clinic under a two week wait [7]. We favor a straight to LAT approach as the yield from cytology for MPM is reported to be low [4], [11]. We use rigid thoracoscopes as we believe the larger working channel allows wider visualization of the pleural cavity, passage of rigid forceps for larger biopsy specimens, and the ability to perform adhesiolysis [6]. As seen in this cohort of patients, LAT with a rigid thoracoscope achieved high diagnostic sensitivity.

The true incidence of “nonspecific pleuritis” (NSP) in our cohort was 14 out of 105 cases analyzed (13%). NSP is an umbrella concept for inflammation or fibrosis of the pleura found in biopsies, which cannot be attributed to a specific benign or malignant etiology [12]. This incidence is lower than in most reported studies, where the incidence of NSP post LAT biopsy is approximately 31% [12]. The lower incidence of NSP is probably due to the retrospective nature of our series, including an extensive review of notes and investigations. In our institution, all patients with BAPE and NSP are followed-up for two years with repeat chest radiographs at six months and 24 months and a CT-scan at 12 months. Thus, it was possible to ascribe *a posteriori* a diagnosis to the patients, even if, at the time of the initial presentation, no specific diagnosis was made. One patient presented with persisting left-sided, lymphocyte-rich pleural effusion, which caused concern for underlying malignancy. This patient was in poor general condition with hemodialysis, chronic obstructive pulmonary disease, unstable type-2 diabetes mellitus, and bilateral leg amputations. A wait-and-see approach was thought appropriate with no biopsies taken during LAT. The macroscopic aspect of the pleura looked normal, and the effusion was drained. Two years later, at the time of writing, the patient has not developed a pleural malignancy.

Usually, at the time of LAT, no histology is available yet. The operator decides to perform talc poudrage based on the macroscopy of the pleura, the clinical and radiological picture, and the presence of a nonexpandable lung. In our cohort, talcum pleurodesis was performed in about half of patients, and was successful in 86%. However, 27 out of 275 patients (9.8% of the total cohort), or 34.6% of patients with nonexpandable lung required another procedure, the vast majority of those being the placement of an IPC. We have thus started to combine LAT with IPC in patients where the lung was adherent to the pleural lining, and participate as an investigational center for the upcoming R-TACTIC study. This randomized study will evaluate the potential of the combination of Thoracoscopic Talc Pleurodesis (TTP) and IPC for reducing time in hospital and breathlessness over one month post randomization versus TTP alone [13]. Further analysis of this cohort will help formulate a more concrete management pathway. We have incorporated the recent recommendation of the BTS, with the Covid-19 pandemic, to perform day case thoracoscopy with indwelling catheter placement [14].

In our series, the incidence of major and minor complications was rare with 5.4%, and 2.5%, respectively, which is in line with the literature studies [2]. In accordance with published national guidance [15], our policy was to give preoperative antibiotics, usually gentamicin. After one lethal complication, our practice and policy have changed, and either flucloxacillin or teicoplanin are now given preoperatively. Three patients (1%) had a pleural space infection, and four (1.4%) had cellulitis around the operation site within the first 30 days following LAT. These seven patients accrued 45 inpatient bed days between them (mean 6.4 days, range 3–10). One of these patients had a positive blood culture (*Streptococcus faecalis*) and required cardiothoracic intervention. The morbidity and length of stay associated with LAT-associated infections support the practice of preoperative antibiotics.

A single patient with mesothelioma developed tumor extension out of the port site, requiring a wide local excision of the painful nodule as well as radiotherapy to the area. Such tumor invasion of the thoracic wall is a known complication of thoracoscopy for MPM with associated morbidity and further treatment needed, but fortunately, the incidence is low [2].

Nine patients (3.4%) patients had prolonged air leaks (PALs) after LAT. We considered PAL as a leakage as lasting longer than five days postoperatively, which is a rather conservative definition [15]. PAL results from air escaping from the lung parenchyma into the pleural space, so the lung parenchyma must have been damaged [16]. However, in our records, there was no single mention of visceral and diaphragmatic pleural biopsy. PALs increase indeed the length of hospital stay [17]. In our cohort, nine patients with PAL accrued 70 inpatient bed days (mean 7.8 days, range 6–14). PALs are associated with pre-existing lung disease and frequently occur in the nonexpandable lung [17]. In our cohort, no patient with PAL had underlying lung disease, but seven patients had trapped lung. At the beginning of our practice, patients with PAL received suction from the wall for a mean duration of three days (range 3–7 days) to re-expand the lung, which might have worsened the leak [16]. Currently, we do not now apply suction after LAT anymore to patients with trapped lung.

We perform LAT under target-controlled anesthesia (TCA) [18], since LAT is a painful procedure [19]. We felt that a combination of midazolam and fentanyl did not provide enough anesthesia and analgesia. Moreover, over-sedation in LAT has been reported in the literature studies [20] and triggers an automatic incident form {DATIX} as part of NHS England’s safety framework [21]. None of the patients offered TCA have required an artificial airway, mechanical ventilation, or intensive care. Against this framework, we have found that sedation and intraoperative anesthesia has been vastly improved since we changed our practice to remifentanyl and Propofol.

One might argue that the monitoring and effort for LAT are similar to as for a “full-size” video-assisted thoracoscopy (VAT). However, LAT has the benefits of not requiring single lung ventilation, reduced pain, and shorter hospital stays [22]. Diagnostic yields are broadly similar, and differences in complications are not statistically significant [23], [24]. However, VATS should be preferred to LAT in difficult pleural cases with adhesions and loculations. Moreover, VATS is more likely to offer up a definitive diagnosis (43.8% of LAT cases showed nonspecific inflammation as compared to only 24.2% of the VATS cases) [24].

This is a single-center, retrospective experience with a high rate of MPM, so that our findings cannot be widely generalized. There was considerable variability in the recording of intraoperative observations on the anesthetic charts. BTS guidance [2] suggests that atrial fibrillation or intraoperative hypotension are minor complications, but we did not collect data on this. However, taken together, our results add to a large body of literature that confirms that LAT, in the hands of experienced physicians, is safe and highly effective at achieving a histological diagnosis. Leaning and adapting practices can be aided by analyses such as the one presented here. Complication rates are low and have improved over time with experience.
